# Imaging in Clinical Trials

**Published:** 2007-05-12

**Authors:** Bradley J Erickson, Jan C Buckner

**Affiliations:** 1 Dept. of Radiology, Mayo Clinic, Rochester, MN;; 2 Dept. of Oncology, Mayo Clinic, Rochester, MN

## Introduction

**Figure 1:**
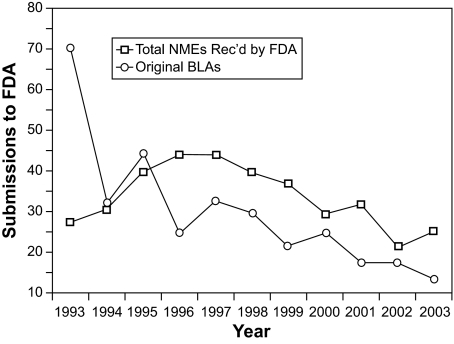
Number of submissions of new molecular entities (NMEs) and biologics license application (BLA) to FDA over the past 10 years. (U.S. Department of Health and Human Services-Food and Drug Administration 2004)

We are experiencing a time of great growth in knowledge about human disease. However, translation of the knowledge into clinical practice has not kept pace. Clinical trials are an important part of the drug development process. The cost of conducting clinical trials has become greater because: 1) regulations on how the trial must be conducted have become more complex; 2) proposed therapies must be compared against standard therapies; and 3) if the end point is survival—it may take longer to reach that end-point as therapies and non-specific supportive measures become more effective. Moreover, therapies administered prior to or subsequent to the experimental intervention may confound the interpretation of survival as an endpoint. Finding valid alternative outcome measures that can be observed soon after the therapy is given could reduce the cost of drug trials, and make effective therapies available to the public more quickly. Imaging can assess therapeutic efficacy for cancers and may be a part of the solution to reduce costs and improve timeliness of clinical trials. (Fig 1).

## The Challenges of Clinical Trials

### Problem 1: Clinical trials are too expensive

**Figure 2:**
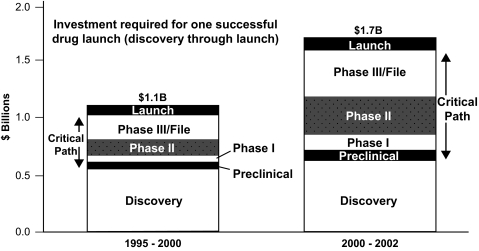
The cost of developing a successful compound is increasing, and the clinical trials pieces are the rapidly increasing components (Windhover’s In Vivo 2003).

Clinical trials are an essential part of the process of documenting the effectiveness of a new therapy. While laboratory experiments attempt to simulate the human situation, validating efficacy and safety in the population of interest remains a necessary step. But the cost of performing a clinical trial large enough to document a treatment effect and monitor for side effects is usually quite expensive. The FDA estimates that the cost to develop a new drug can be as high as $1.7 billion (Fig 2), with others estimating that the median cost at ‘only’ $800 million (DiMasi, 2002).

Some believe it is this mounting cost that is responsible for the decline in the number of new agents being submitted to the FDA. This represents a great challenge to our health care system. No amount of research is going to be effective in curing cancer if the final step of performing the clinical trial is too difficult or expensive to justify the economic returns expected from selling the product. Developing methods to reduce the effort and cost of a clinical trial while maintaining or increasing the validity would be valuable.

### Problem 2: Getting enough patients for a timely trial

Another issue with clinical trials is patient recruitment. Currently, about 3% of adult patients with cancer participate in clinical trials. The reasons are not fully known, but likely include: patient and physician awareness, trial availability, limited eligibility criteria, concern about whether their insurance will cover the costs, and the logistics of participating. Some have argued that since there are few truly curative therapies, nearly all patients are effectively in a clinical trial (Jaffe, 2005) but without the benefit of evidence-based data.

While a dramatic effect is detectable in a small cohort, seeing small effects requires many subjects. While everyone wants a ‘silver bullet’ cure, seeing small effects may be beneficial in setting a general direction that may be fruitful. Furthermore, getting a large homogeneous cohort of patients is essential in accurately defining the benefits of therapy. Diversity in the population and in the tumor itself likely accounts for much of the variability in outcomes.

### Problem 3: Getting enough of the right types of patients

The advance of genetic technology allows separation of the population into prognostically homogeneous groups. It is known that for some diseases, genetic differences in the tumor or in the patient can predict the success of a therapy and may have prognostic value as well. Imaging-based markers could serve this role. For example, if there is genetic variability in drug absorption, transport, or metabolism, the ability to ‘see’ that a given agent is actually localizing to a tumor might predict which patients would or would not benefit from a particular therapeutic approach.

### Problem 4: Mining the data in a clinical trial for all effects

A recent trend in clinical research is to analyze data collected for a certain clinical trial and attempt to find not only the sub-populations for whom a therapy worked well (or poorly), but also to look for unexpected effects that could be useful in applying the therapy to other diseases (Salamone, 2006). In order to do this, assuring proper collection of all data and metadata in a standardized fashion is essential. Being able to ask questions that weren’t known or of interest at the time the data collection process was defined is essential to gleaning all reliable information present in the data set.

## The Possible Solution: Imaging as a Biomarker

In March of 2004, the FDA announced plans to reform the regulatory path to reduce the barrier to new drug development. Imaging was identified as one of the components that could play an important role in reducing the cost of clinical trials, which is a major component of drug approval costs (Mills, 2005). In particular, using time-to-progression (often detected by imaging) rather than survival as the outcome measure can reduce costs and improve timeliness.

Imaging has traditionally played 3 roles that relate to clinical trials: detection, characterization, and monitoring/assessment. The first 2 of these receive the lion’s share of attention in training radiologists and in certification. Developing imaging modalities that can better detect and characterize lesions also receives the lion’s share of grant dollars, with relatively little funding going to the evaluation and validation of imaging-based assessment. Advances in medical imaging have resulted in substantially improved detection and diagnosis of tumors over the past 25 years, but its role in therapy advancement has been minimal.

NCI has issued RFAs focused on development of novel imaging agents and analysis methods for clinical trials to attempt to address that imbalance. This includes funding for the development of molecular agents that may help to determine the distribution of an agent within the body over time, as well as functional imaging methods that may help to demonstrate physiological changes in the tumor or the host that might be associated with clinical benefit.

New endpoints for phase I and II trials focusing on time to progression or progression free survival, commonly determined by imaging are becoming more popular (Seymour, 2002). These seem to correlate well with survival, and can be determined much sooner, allowing a shorter, less expensive trial design.

## Factors that Limit the Role of Imaging in Clinical Trials

### Data collection and sharing

The lack of good mechanisms and motivators for sharing data have minimized the amount of sharing that occurs between trials. Having control groups for every study may be a cost that could be reduced with proper study collaboration and design. One recent demonstration of the potential of collaboration was the Digital Mammography in Screening Trial (DMIST) in which four vendors of digital mammographic equipment combined results to achieve the large recruitment demands in a reasonable period of time (almost 50,000 subjects in 1 year) (von Eschenback, 2005).

The maximum benefit of sharing data is gained when the imaging collected is done in a high quality and uniform way. That means it is critical that imaging experts be involved in the design of the clinical trial which utilizes imaging as an endpoint (Kothari, Guermazi et al. 2003) in order to assure that the most appropriate imaging is used. Most clinical trials require considerable time to complete and using shared data would likely increase the longitudinal value of the data. That means that using the most reliable and accurate image techniques feasible will make the database relevant into the future.

### Standardization of imaging protocols

There are several issues which can arise when using imaging as an endpoint. As an example, dynamic contrast-enhanced MRI has become a popular method for measuring perfusion. In addition to the usual exclusion of patients who can’t have an MRI, good perfusion imaging requires good cardiac output and the ability to place a large venous catheter. The MRI scanner itself must also be of reasonably recent vintage. There may also be variations in the appearance of images between brand of scanner and software levels. Perfusion imaging works better in some body parts than others. Perfusion imaging usually does not produce an absolute value, but rather a value expressed as a ratio to some normal structure—something which may be easier for body parts like brain than for others like prostate. Having practical experience with imaging will help to sort out feasible imaging methods for the setting planned (e.g. tertiary academic center versus community setting).

Many clinical trials were conducted with the maximum feasible patient privacy, but this was often difficult with film images. For many studies, the clinical films were copied, and then the patient identifiers were obliterated using permanent markers, and replaced with study identifiers. This was often a less-than perfect process.

As mentioned previously, it is becoming increasingly important to document the collection of data, and that the data have a verifiable audit trail. The DICOM (DICOM Standards Committee) (Digital Image Communications in Medicine) standard facilitates this by embedding unique identifiers into each image. It also has added security mechanisms for secure transport and audit trails. However, it has not included watermarking technologies because these require alteration of pixel data—something that is generally to be avoided. Some other image integrity method might be considered for images, but DICOM provides a good base on which to build.

### Standardized Image Review and Assessment

Because the changes in imaging studies are often subtle, and because consistency in decisions about changes in tumor status are critical to the interpretation of trial results, having standardized image assessment is critical. This means that image data ideally is interpreted consistently by experienced reviewers. In the past, this was done by making copies of films, sending them to the central site, which had to store these, then provide a ‘reading environment’ for assessment, and manual transfer of measurements into the clinical trials database. If there was more than one reader, this might be replicated, and if that reader was from a different site (an important part of validation) either the reader would have to come to the site, or all the films would have to be sent to the other reader. This was an expensive process, making central review the exception rather than the rule for clinical trials.

It was noted above that it is critical that images for clinical trials be collected using uniform image acquisition methods to minimize the variability. Unfortunately, there will be some variability due to proprietary differences in commercial imaging equipment. This will require either that one brand and model of imaging equipment be selected for each clinical trial, or that standardization methods be developed so that differences across vendors and models can be eliminated. The American College of Radiology has initiated UPICT—the Uniform Protocols for Imaging Clinical Trials with the expressed goal of developing “..widely acceptable consistent imaging protocols and quality control procedures across the multiple sites and modalities needed for case accrual…”(The American College of Radiology, 2006).

Standardized image measurement methods are also critical. The complexity of doing a measurement well may be responsible for the fact that the most accepted response assessment method for imaging uses a single dimension measurement (Therasse, Arbuck et al. 2000). Applying statistical methods to reduce variability in reader assessment of therapies with standardized tools could allow a 60% reduction in the size of the cohort required for assessing some devices (Wagner and Beiden 2003; Wagner, Beiden et al. 2003). Similar reductions seem feasible for non-device therapies.

Electronic image management has the potential to improve the quality of data analysis while reducing the cost. In addition to automating the collection of measurements (which should reduce errors and human effort), distributing and managing digital image data and measurements is likely more reproducible, faster, and less expensive than film-based methods.

## Integration of Imaging into Cancer Informatics

### Metadata standardization

Metadata is the data that describes the data of interest, in this case, the image data. Imaging meta-data includes information such as the device used, the settings used, contrast agents, and possibly any processing of the data. This information is critical to image appearance, and therefore, to the proper conduct of a clinical trial. Getting agreement on the basic metadata in imaging is as contentious as it is for the other parts of the medical record. There is a good standard for much of imaging metadata, which is DICOM. The good news that there is a standard is also a challenge—it is difficult for a standard to keep pace with the rapid pace of technology, and clinical trials often use the newest methods. The bad news is, one must often develop non-standard ways to represent pieces of the metadata that DICOM cannot address.

### Metadata collection and integration into clinical trials database

Integration of imaging metadata into the clinical trials database may be valuable for the conduct of clinical trials because it allows easier assessment of variations in imaging methods. While the ideal study would have everything identical except for whether or not a therapy was given, such a requirement would likely make the study impractical in a multi-institutional setting. This is particularly true as one contemplates the large studies potentially occurring in communities as a part of routine clinical care. In that case, being able to segregate certain populations based on the scanning protocol could help to determine whether the scanning protocol used has an effect on the trial.

### Quality review process

The importance of standard image acquisition was emphasized above. That is one part of the quality control process. Other critical elements are that the correct patient is being studied. This can be a challenge with strong privacy requirements. It is also important to assure that the protocol validated at one location is actually executed at all sites. This typically requires involvement of a medical physicist who can will help to develop the protocol itself and then to develop procedures that will validate the protocol on all other sites.

Standardization of the imaging protocol can permit greater automation of the image acceptance process. The first step is verifying that the protocol was followed. With good metadata, this can be partly automated. For example, use of the proper TR/TE or kVp can be determined; but also, the signal-to-noise ratio should be fairly well controlled, and could be automatically measured. However, other critical aspects of quality control include: validating that the whole anatomic field was included; that the patient did not move so much as to compromise the study; and that any external methods (e.g. contrast administration) were handled correctly. These matters typically require human visual assessment. Communicating any failure to adhere to the protocol rapidly can reduce the chance of losing a valuable data point and usually improves adherence to the protocol.

Even if the imaging device is operated properly, the images may not be acceptable. The patients are often ill both due to the disease, and due to effects of therapy. This can result in an inability to hold still for good images. Determining when the data degradation due to motion, patient artifacts, or other problems, is greater than degradation due to loss of a data point is a challenge that requires human judgment in most cases.

Finally, characterizing the statistical properties of the measurement method is also essential to predict the power of a study. For categorical data, one can use kappa statistics or percentage agreement. Weighted kappa statistic or Spearman’s *rho* can be used for ordinal data. For continuous data or geometrical measurements, one may use correlation coefficient, Pearson correlation coefficient, or intra-class correlation coefficient. These measures must be determined for each measurement method and software for each body part, modality, and perhaps vendor and software level on the scanner.

Measuring deterministic processes is easier, and should produce much less variation. These should be preferred over stochastic or less predictable methods where feasible. A human operator can be a source of substantial measurement variability. High quality longitudinal databases could allow characterization of different assessment methods and selection of those methods (and human operators) that have the least variability in producing measurements.

## Conclusions

Standardization of imaging acquisition, metadata reporting, electronic data management, and image interpretation/quantification will improve the accuracy of endpoint determination in clinical trials. Once validated, these improvements in image-dependent endpoints may reduce the costs and time to complete clinical trials.
